# Exploring the Integration of Occupational Therapy in Pediatric Oncology Care in Spain: A Descriptive Study

**DOI:** 10.3390/healthcare13141737

**Published:** 2025-07-18

**Authors:** Sandra León-Herrera, Elisabet Huertas-Hoyas, Raquel Gómez-Bravo, José María Fraile Vicente, Elisa Bullón-Benito, Mª Pilar Rodríguez-Pérez

**Affiliations:** 1 Department of Psychology and Sociology, University of Zaragoza, 50009 Zaragoza, Spain; sleon@unizar.es; 2 Department of Physiotherapy, Occupational Therapy, Rehabilitation and Physical Medicine, Rey Juan Carlos University, 28922 Alcorcón, Spain; elisabet.huertas@urjc.es (E.H.-H.); elisa.bullon@urjc.es (E.B.-B.); pilar.rodriguez@urjc.es (M.P.R.-P.); 3 Rehaklinik, Centre Hospitalier Neuro-Psychiatrique (CHNP), 9012 Ettelbruck, Luxembourg; 4 Palliative Care Unit, Jimenez Diaz Foundation University Hospital, 28040 Madrid, Spain; josemariafraile@gmail.com

**Keywords:** occupational therapy, pediatric oncology, multidisciplinary care, cancer rehabilitation, Spain

## Abstract

**Background/Objectives**: Childhood cancer leads to significant physical, cognitive, and psychosocial consequences that adversely affect the development and quality of life. Occupational Therapy (OT) has the potential to mitigate these effects. However, its integration into pediatric oncology care in Spain remains limited and underexplored. This study aims to examine the availability, characteristics, and perceived impact of OT services within pediatric oncology units across Spain and to identify key barriers to their implementation. **Methods**: A descriptive, cross-sectional study using a mixed-methods approach was conducted. An online questionnaire was distributed to healthcare professionals working in pediatric oncology units nationwide. Quantitative data were analyzed using descriptive statistics, Fisher’s exact test, and odds ratios with 95% confidence intervals to explore associations. Effect sizes were calculated using Cramér’s V where applicable. Qualitative responses underwent inductive thematic analysis. **Results**: A total of 42 hospital centers from 12 autonomous communities participated. Only 16 reported having OT services in pediatric oncology, with notable regional disparities. A significant proportion of respondents were unaware of the integration of OT in their institutions. Identified barriers included lack of resources, insufficient specialized training, and limited institutional recognition of OT. Nonetheless, professionals familiar with OT interventions reported positive outcomes, particularly in improving patients’ functional autonomy, emotional well-being, and social participation. **Conclusions**: OT remains insufficiently integrated into pediatric oncology care in Spain. To optimize the quality of care, it is essential to address educational, structural, and institutional challenges and promote OT as a key component of multidisciplinary teams.

## 1. Introduction

Childhood cancer, although relatively rare, presents a significant public health challenge due to the complexity of its treatment and its long-term impact on development. In Spain, the most common pediatric cancers include leukemia (27.5%), central nervous system tumors (26.4%), and lymphomas (12.3%) [1,2,3].

Globally, the World Health Organization estimates that approximately 400,000 children and adolescents are diagnosed with cancer each year [4]. In 2023, the Spanish Registry of Childhood Tumors (RETI-SEHOP) reported 993 new cases among children aged 0–14, contributing to a total of 9202 cases between 2020 and 2023 [2,5].

While childhood cancer has a lower mortality rate compared to adult cancer [6], it often leads to significant developmental and functional consequences. These vary widely depending on the diagnosis and treatment received and may include physical impairments (e.g., neuropathies, motor difficulties, pain), cognitive deficits (e.g., attention, memory, executive functioning), and psychosocial challenges that affect both mental health and social integration [1,7,8,9]. As a result, children’s participation in daily activities and their long-term quality of life can be seriously impacted.

In this context, a multidisciplinary rehabilitation approach, in which Occupational Therapy (OT) may play a key role, is crucial to promoting autonomy, enhancing recovery, and supporting reintegration into everyday life [8,10].

Although OT is commonly present in early intervention centers with a family-centered approach, its presence in pediatric oncology remains limited. According to the World Federation of Occupational Therapists, OT aims to enable individuals to participate in everyday activities by enhancing their abilities or adapting environments to support occupational engagement [11]. These core objectives align closely with the complex and evolving needs of children undergoing cancer treatment.

The Professional Association of Occupational Therapists of Navarra highlights the importance of OT across all phases of the cancer journey—diagnosis, treatment, and survivorship—through interventions addressing fatigue, pain, emotional adjustment, daily activity performance, and social reintegration [12]. While the literature on OT in pediatric oncology in Spain remains scarce, existing studies suggest that OT and interdisciplinary rehabilitation approaches contribute positively to functional outcomes and quality of life in oncology patients [13,14,15].

Despite the growing number of occupational therapists in Spain—8450 registered in 2023 [16]—data on their involvement in oncology care, especially pediatrics, are limited. A 2019 national survey identified just 612 therapists working in the public health system, with notable regional disparities and little clarity regarding their areas of practice, particularly in pediatric settings [17].

Due to the potential benefits of OT in pediatric oncology and the current lack of data on its integration in Spain, this study aims to examine the availability, characteristics, and perceived impact of OT services within Spanish pediatric oncology units, as well as to identify the main barriers to their inclusion in multidisciplinary teams.

## 2. Materials and Methods

### 2.1. Study Design

This is a descriptive, cross-sectional study with a mixed-methods approach (quantitative and qualitative) aimed at exploring the integration of OT in pediatric oncology care in Spain. Data were collected through an online questionnaire combining closed-ended (quantitative) and open-ended (qualitative) questions to gather comprehensive insights into the availability, implementation, training, and perceived impact of OT services in pediatric oncology units.

### 2.2. Participants

#### Inclusion and Exclusion Criteria

Participants in this study were selected based on specific inclusion and exclusion criteria to ensure the relevance and reliability of the data. Eligible participants included health professionals such as occupational therapists, pediatric oncologists, unit coordinators, psychologists, nurses, and other members of multidisciplinary teams, all of whom were actively working in pediatric oncology units within hospitals in Spain, whether public or private. A minimum of 6 months of professional experience in pediatric oncology was required to ensure sufficient familiarity with the clinical context and institutional practices. Additionally, participants were expected to have a comprehensive understanding of the structure, organization, and functioning of pediatric oncology services in their respective institutions.

Professionals were excluded from the study if they were not currently working in the field of pediatric oncology, were employed outside of Spain, or had less than 6 months of relevant experience. Likewise, individuals who did not have direct clinical involvement with pediatric oncology patients were not considered eligible for participation.

At the beginning of the survey, participants were asked to provide verification details regarding their professional role, hospital location (region and public/private status), and their experience in pediatric oncology. Inconsistent or ineligible responses were reviewed and excluded by the research team.

### 2.3. Recruitment

Participants were recruited through institutional emails sent to pediatric oncology unit coordinators or other key professionals in hospitals across Spain. When contact information was not available online, the team called the hospital directly to obtain the appropriate email addresses. Formal approval was requested from each hospital before disseminating the survey, ensuring compliance with institutional protocols and ethical standards.

Once approval was granted, the survey link was sent via email to identify key informants. The message included detailed information about the research objectives, confidentiality assurances, and instructions for completion. Distribution support from unit coordinators ensured that eligible professionals received the survey.

### 2.4. Sample Size and Sampling Strategy

Due to the exploratory nature of the study, the sample was not intended to be statistically representative. Instead, a purposive key informant sampling strategy was used to obtain in-depth insights from professionals with direct experience in pediatric oncology care. The initial objective was to recruit a minimum of 12 hospital centers, selected to ensure diversity in geographic location, institutional structure (public and private), and levels of service provision across Spain’s autonomous communities.

This sampling approach was designed to capture a broad range of institutional contexts and variations in the availability and integration of OT services. By including hospitals of different sizes and resource levels, the study aimed to develop a nuanced understanding of the barriers and facilitators associated with OT implementation in pediatric oncology settings.

### 2.5. Data Collection

Data were collected through an online questionnaire created and administered using LimeSurvey, a secure and GDPR-compliant platform. The questionnaire included closed-ended questions (multiple choice, Likert scales) for quantitative data and open-ended questions to allow professionals to elaborate on their perceptions and experiences. Data collection occurred over a 3-month period. The instrument explored availability and characteristics of OT services, training of professionals, types of interventions, institutional barriers, and perceived patient outcomes. The complete survey is available as Supplementary Materials.

To ensure the validity of the instrument, the survey was reviewed by two independent experts in pediatric oncology prior to distribution. They evaluated the content for face validity, assessing the relevance, clarity, and appropriateness of the questions for a multidisciplinary respondent group. Minor revisions were made based on their feedback to enhance clarity and usability.

### 2.6. Variables Collected

The questionnaire was designed to collect detailed, multi-dimensional information regarding the presence, characteristics, and impact of OT services within pediatric oncology settings in Spain. It was structured into thematic sections that addressed both structural and experiential aspects of care, from institutional availability to professionals’ perceptions of effectiveness.

First, data were gathered on the availability of OT services, including whether OT was offered in the hospital or unit, how long the service had been in place, how many therapists were employed, and at which stages of cancer treatment (e.g., diagnosis, active treatment, remission, palliative care) OT interventions were provided. Additionally, the settings in which therapy occurred (inpatient, outpatient, specialized units) were also explored.

The second dimension focused on the training and expertise of occupational therapists, assessing the extent of their specific education in pediatric oncology, whether their training was deemed sufficient, and how frequently they engaged in ongoing professional development such as seminars or congresses.

A substantial section was devoted to the nature of OT interventions, capturing which domains were prioritized, ranging from physical rehabilitation and ADL (activities of daily living) support to emotional adjustment, social participation, and cognitive rehabilitation. Respondents were also asked about how interventions were prioritized, if they were personalized based on patient characteristics (e.g., age, type of cancer), and whether the work was conducted collaboratively with other disciplines.

The questionnaire also examined barriers and challenges to OT integration. This included institutional or systemic obstacles such as lack of funding, limited staffing, low recognition from hospital leadership, interprofessional coordination issues, and difficulties in patient referral. Insight was also gathered on the perceived adequacy of time and resources for delivering quality care.

Finally, the survey explored the perceived impact of OT on patient outcomes. Professionals were asked to evaluate the role of OT in enhancing mobility, promoting independence in daily life activities, and supporting psychosocial well-being. Moreover, they were prompted to reflect on whether OT services were seen as essential, how satisfied patients and families were, and what improvements they had observed as a result of therapy.

A summary of the key variables collected is shown in Table 1.

### 2.7. Data Analysis

#### 2.7.1. Quantitative Analysis

Quantitative data were analyzed using IBM SPSS Statistics (version 29). Descriptive statistics, including frequencies and modes, were used to summarize the characteristics of participating centers and the availability and structure of OT services. The significance level was set at α = 0.05 for any applicable statistical tests.

Bivariate analyses using Fisher’s exact test were conducted to explore potential associations between institutional factors (such as hospital type, number of patients treated, and geographic location) and the presence or characteristics of OT services.

To evaluate the strength of associations, effect sizes were calculated using Cramér’s V, with 95% confidence intervals reported where applicable. Responses of “Don’t know” were treated as missing data and excluded from statistical analyses.

#### 2.7.2. Qualitative Analysis

Open-ended responses were analyzed using inductive thematic content analysis with the support of NVivo software (version 15). The analysis followed Braun and Clarke’s six-phase framework, which includes familiarization with the data, generation of initial codes, identification and review of themes, theme definition, and final reporting.

To ensure analytical rigor, coding was performed independently by two researchers. Intercoder reliability was assessed using percent agreement, which reached 82%. Discrepancies between coders were discussed and resolved through discussion and consensus meetings. This process helped strengthen the trustworthiness and consistency of the thematic findings. Data saturation was determined when no new relevant themes emerged.

### 2.8. Ethical Considerations

Participation in the study was entirely voluntary and anonymous. The online questionnaire included a detailed information sheet outlining the objectives, confidentiality measures, and data protection procedures. Informed consent was implied by survey completion. All responses were anonymous, and data were stored securely in compliance with data protection regulations. Only the research team had access to the raw dataset.

The study received ethical approval from the Clinical Research Ethics Committee of Aragón (CEICA), under the code: C.I. PI25/055.

## 3. Results

### 3.1. Quantitative Results

#### 3.1.1. Availability and Characteristics of OT Services

A total of 42 hospital centers from 12 autonomous communities across Spain responded to the survey, although the number of valid responses varied by item due to missing data.

The distribution of respondents by profession was as follows: 35.7% were occupational therapists, 28.6% were pediatric oncologists, 16.7% were psychologists, 11.9% were nurses, and 7.1% were other healthcare professionals, such as coordinators or allied health staff. This multidisciplinary composition reflects the range of perspectives involved in pediatric oncology care across the participating hospitals.

Table 2 presents general characteristics of the participating hospitals, including type, geographic distribution, annual patient volume, and age groups served. Most were public institutions (83.3%), with the highest representation from the Community of Madrid (21.4%). Approximately 35.7% of centers reported treating between 50 and 100 pediatric oncology patients annually, with children and adolescents aged 6 to 18 years being the most commonly served age group.

Table 3 summarizes data specific to the presence and characteristics of OT services. Only 16 centers reported having OT in pediatric oncology units. Among these, most had provided such services for more than 5 years, typically with staffing levels between two and five occupational therapists. This reflects a markedly uneven distribution and structure of OT support across the surveyed institutions.

#### 3.1.2. Training and Expertise

Responses regarding the knowledge and training of occupational therapists in pediatric oncology revealed significant limitations. As shown in Table 4, knowledge about specific oncology treatments (e.g., chemotherapy, radiotherapy, and transplants) was low: only 7.1% of therapists rated their knowledge as high, while an equal percentage acknowledged either low or no knowledge. Furthermore, 9.5% selected “Do not Know” (DK), suggesting widespread uncertainty or lack of familiarity with oncology-specific concepts, and 69% did not even answer the question. The large amount of missing data (non-responses) may be explained by the fact that, in hospital centers without an occupational therapist, respondents were not permitted to answer certain training-related questions.

Formal oncology-specific training, encompassing treatment protocols, side effects, and patient-specific needs, was also limited. Only 38.5% of respondents reported receiving such training, while nearly half (11.9%) did not. A majority (59.5%) believed that more specialized education in this area is necessary, with no participants disagreeing, although 16.7% expressed uncertainty.

Perceptions of the sufficiency of current training were also mixed. Just under one-third (9.5%) felt adequately prepared to address the needs of pediatric oncology patients, while over half (16.7%) were uncertain. Similarly, participation in ongoing professional development was inconsistent, with only one-third of centers reporting engagement in continuing education programs specific to pediatric oncology. These data underscore the need for systematic efforts to enhance the clinical competencies of occupational therapists in this domain.

#### 3.1.3. OT Interventions

**Figure 1:**
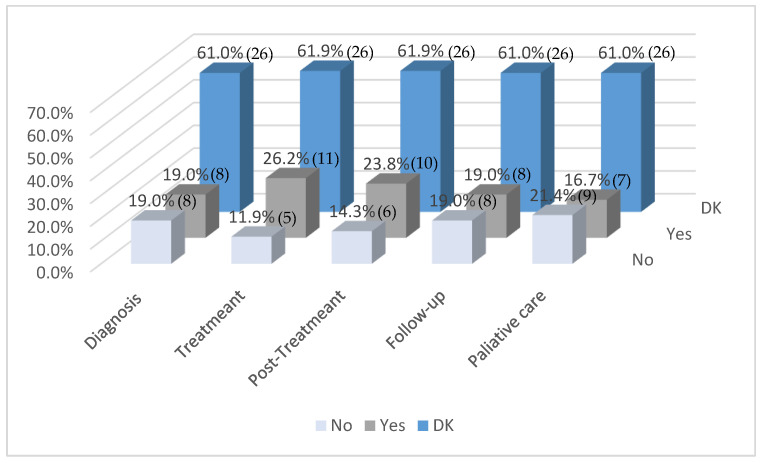
Occupational therapists included in the different phases of treatment (*n* = 42). Note: Values in parentheses indicate the number of respondents (*n*) corresponding to each percentage.

Figure 1 illustrates the reported inclusion of occupational therapists across various phases of pediatric oncology care. A significant proportion of respondents (61.9%) consistently selected DK for all phases—diagnosis, treatment, post-treatment, follow-up, and palliative care—indicating a high level of uncertainty or lack of information regarding the integration of OT within their institutions.

Among those who did provide definitive responses, OT was most frequently reported as integrated during the treatment (26.2%) and post-treatment (19%) phases. Lower levels of inclusion were noted during the palliative care (16.7%), follow-up, and diagnostic (19%) phases. Notably, a non-negligible percentage of respondents indicated that OT was not included in any phase, particularly in palliative care (21.4%), follow-up, and diagnosis (19%).

Overall, the data suggest limited and inconsistent integration of OT throughout the pediatric oncology care continuum, compounded by considerable gaps in knowledge about its implementation.

**Figure 2:**
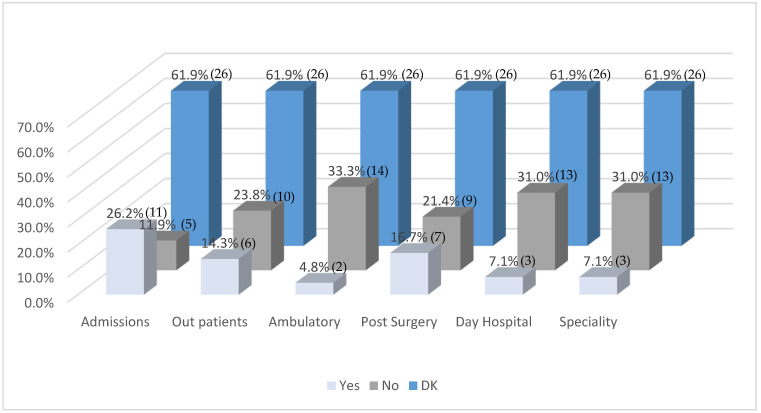
Areas of intervention of the occupational therapist in the oncology patient (*n* = 42). Note: Values in parentheses indicate the number of respondents (*n*) corresponding to each percentage.

Figure 2 presents data on the areas of care in which occupational therapists are involved in the treatment of oncological patients. As with the previous figure, a substantial proportion of respondents (61.9%) selected DK across all care settings, reflecting a widespread lack of information regarding the scope of OT interventions in these clinical contexts.

Among respondents who did indicate specific areas of intervention, the highest levels of inclusion were reported in admissions (26.2%), followed by post-surgery care (16.7%) and outpatient consultations (14.3%). In contrast, OT was reported to be rarely involved in ambulatory care (4.8%), day hospital (7.1%), and specialty services (7.1%) despite their relevance in pediatric oncology. Conversely, some areas showed relatively higher exclusion rates, particularly ambulatory care (33.3%), day hospital, and specialty services (31%), suggesting limited integration of OT in these domains.

These findings highlight both the uneven distribution of OT services across different healthcare settings and a pervasive uncertainty among respondents, which may indicate systemic gaps in interprofessional collaboration or institutional awareness regarding the role of OT in oncology care.

#### 3.1.4. Barriers and Challenges

**Figure 3:**
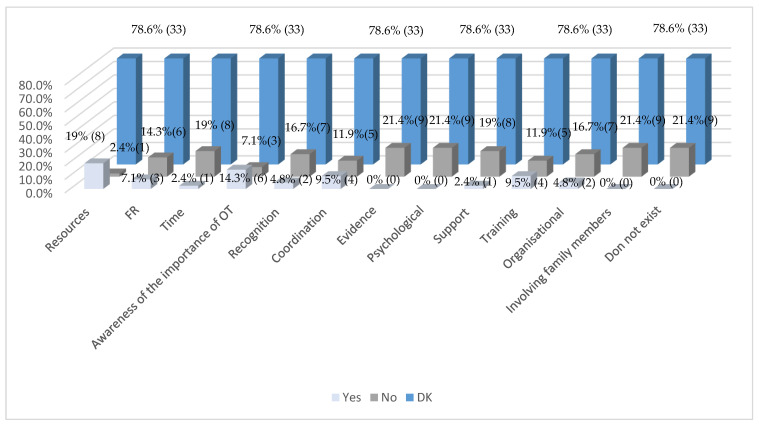
Main obstacles to the integration of OT in pediatric oncology care in your centre/unit (*n* = 42). Note: Values in parentheses indicate the number of respondents (*n*) corresponding to each percentage.

Regarding the identification of the most frequent barriers to the integration of OT in pediatric oncology care within each center or unit, 78.6% of respondents reported not knowing with certainty which factors might be hindering the broader incorporation of this discipline. Among the barriers that were identified, the most commonly cited were: lack of resources (personnel, space, materials) (19%), limited awareness of the relevance of OT (14.3%), and insufficient training and coordination between healthcare services (9.5%) (Figure 3).

In contrast, several factors were not perceived as significant obstacles to OT integration. These included: lack of time among healthcare professionals due to workload (19%), lack of recognition of OT’s value by the multidisciplinary team (16.7%), poor coordination between healthcare services (11.9%), limited evidence of OT’s effectiveness in this context (21.4%), emotional or psychological barriers in patients (e.g., fear or rejection of treatment) (21.4%), resistance or lack of support from other team professionals (19%), insufficient training of healthcare personnel (11.9%), organizational or administrative barriers (16.7%), and difficulties involving the family in treatment (21.4%). Additionally, 21.4% of respondents stated that there are no barriers to the integration of OT.

These findings highlight both the variability and limitations in the implementation and perception of OT in pediatric oncology across Spanish hospitals.

#### 3.1.5. Perceived Impact

**Figure 4:**
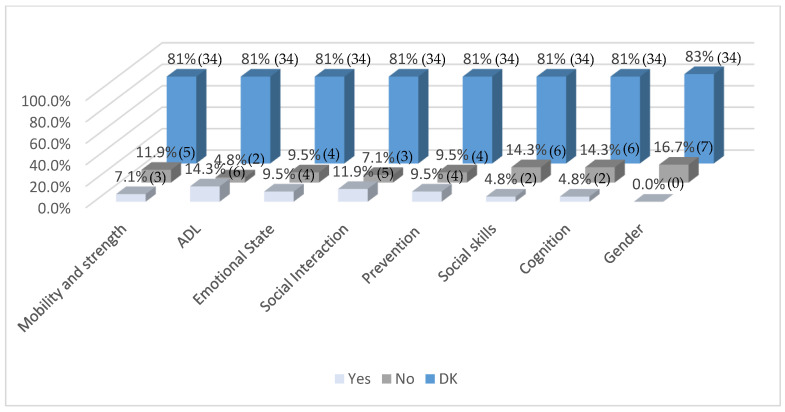
Areas in which OT has the greatest impact on pediatric oncology patients in your centre/unit (*n* = 42). Note: Values in parentheses indicate the number of respondents (*n*) corresponding to each percentage.

Figure 4 illustrates the perceived areas of greatest impact of OT in pediatric oncology patients, as reported by participating centers. A notable finding is that 81% of respondents selected DK across all evaluated domains, highlighting a widespread lack of clarity or information about the specific contributions of OT in this clinical context.

Among those who provided affirmative responses, activities of daily living (ADL) (14.3%), social interaction (11.9%), emotional state (9.5%), and prevention (9.5%) were the most frequently recognized domains where OT had a positive impact. Lower impact was reported in areas such as mobility and strength (11.9%), social skills (14.3%), cognition (14.3%), and gender-specific considerations (16.7%).

These results suggest limited recognition of OT’s role in both physical and psychosocial domains within pediatric oncology care, and they underscore the need for greater dissemination of evidence-based practices and interdisciplinary collaboration to enhance the visibility and perceived value of OT interventions.

#### 3.1.6. Associations Between Institutional Characteristics and OT Presence

For the 2 × 2 analysis, the OT presence variable was recoded as “yes” versus “no/do not know,” grouping the latter two as indicating no explicit statement. This approach was chosen to facilitate comparison and maximize sample size. Hospital type was recoded as “public” versus “non-public.” Fisher’s exact test was used for the analysis, and odds ratios (ORs) with 95% confidence intervals (CIs) were calculated. Similarly, the patient volume variable was regrouped into “up to 100” and “over 101.”

No statistically significant associations were found between hospital type (public vs. non-public) and OT service availability (*p* = 0.546; OR = 0.788; 95% CI: 0.152–4.08), nor between patient volume and OT presence (*p* = 0.530; OR = 1.613; 95% CI: 0.434–5.99). However, descriptive data suggest a tendency for larger, public institutions to report greater availability of OT resources.

### 3.2. Qualitative Results

An inductive thematic analysis was conducted on the open-ended survey responses provided by the healthcare professionals working in pediatric oncology. The aim was to explore their perceptions of the role, benefits, and areas for improvement of OT within this clinical context. Five main themes emerged from the data, each reflecting key aspects of the respondents’ experiences and perspectives.

#### 3.2.1. Perceived Impact of OT on the Reduction of Treatment Side Effects

A dominant theme was the significant positive impact attributed to OT in mitigating the side effects of cancer treatment. Participants frequently highlighted improvements in symptoms such as fatigue, pain, and motor impairments.

*“Very positive, it significantly reduces the side effects of treatment.”*

*“It helps manage pain and improves the child’s emotional well-being.”*

#### 3.2.2. Observed Improvements Following OT Interventions

Three key areas of improvement were identified in relation to patient outcomes:

- Functional autonomy: enhanced ability to perform activities of daily living.
- Social integration: increased interaction and participation with peers.
- Overall quality of life: improvements in physical and emotional well-being.

*“They become more autonomous and integrate more easily with other children.”*

*“They have improved in mobility, emotional state, and cooperation.”*

#### 3.2.3. Inclusion of OT in Standard Care Protocols

Responses revealed divergent views on whether OT should be systematically included in pediatric oncology care pathways:

- Split perspectives: while some professionals support its integration into standard protocols, others expressed reservations.
- Conditional applicability: the perceived need for OT varied depending on patient characteristics and the stage of treatment.

*“It would be ideal to integrate it into care protocols from the time of diagnosis.”*

*“In most cases, I don’t find it necessary, although it can be useful for specific patients.”*

#### 3.2.4. Areas for Improvement in OT Practice

Participants identified several key areas where OT services could be strengthened:

- Specialized training: a need for more focused education in pediatric oncology for occupational therapists.
- Interdisciplinary integration: better incorporation of OT professionals within multidisciplinary teams.
- Resources and frequency: limited human and material resources were noted as barriers to consistent intervention.

*“More training and courses to raise awareness of its importance.”*

*“Interdisciplinary integration and more resources in public hospitals.”*

*“There is a lack of awareness about the added value that occupational therapy provides.”*

#### 3.2.5. Additional Comments

Two recurring ideas emerged from additional comments provided by participants:

- Partial lack of awareness: some professionals acknowledged limited knowledge about the role of OT in pediatric oncology.
- Recognition of value: others emphasized the discipline’s essential contribution to patient recovery.

*“It is a fundamental discipline in the recovery of the child.”*

*“It would be useful to promote more research in this field.”*

Overall, the qualitative findings reflect a generally positive perception of OT in pediatric oncology, particularly in terms of enhancing functional performance and quality of life. Nonetheless, clear needs remain in areas such as professional training, interdisciplinary collaboration, and resource allocation. Addressing these challenges represents an important opportunity to strengthen the role of OT as a core component of comprehensive pediatric cancer care.

## 4. Discussion

This study aimed to explore the integration of OT in pediatric oncology care in Spain, an area of growing international relevance but limited development within the national context. The findings reveal notable disparities in OT service availability, limited specialized training, and a widespread lack of institutional recognition, underscoring the need for systemic and educational reforms.

Despite increasing recognition of the importance of interdisciplinary rehabilitation for pediatric cancer patients [8], our results suggest that OT remains underutilized in Spanish pediatric oncology units. Only 16 out of 42 centers reported having OT services, and a significant proportion of respondents selected “I do not know” when asked about the phases of cancer care in which OT was involved. This not only reflects a limited structural presence of OT but also highlights a widespread lack of knowledge or institutional awareness regarding its role and scope in pediatric oncology.

This knowledge gap is not unique to Spain. Previous studies have reported similar findings internationally, where OT is often underrecognized by both healthcare providers and policymakers in oncology services [18,19]. A scoping review by Wallis et al. (2020) identified diverse OT roles in cancer care but noted a lack of evidence for treatment outcomes, especially for children and adolescents [18]. In the Spanish context, OT remains poorly integrated into hospital systems, especially in oncology, where its inclusion in interdisciplinary teams remains limited [20]. To address these challenges, several authors emphasize the need for enhanced interprofessional collaboration, particularly between occupational therapists and nursing professionals, as well as the promotion of OT’s presence in oncology and palliative care pathways, through greater investment in education, research, and institutional support [20,21].

This limited recognition was also reflected in both the quantitative and qualitative data. In the quantitative analysis, 53% of participants were unable to identify any clear barriers to OT integration—an indirect indicator of low visibility or limited understanding of the discipline. Notably, 9.5% explicitly cited lack of knowledge about OT as a key obstacle. Qualitative responses reinforced this finding, with many professionals admitting to minimal awareness of OT’s role in pediatric oncology, even while acknowledging its potential benefits.

These findings contrast with international evidence highlighting the value of OT in improving outcomes for pediatric oncology patients, including enhanced functional autonomy, emotional well-being, and overall quality of life [22,23]. In our study, professionals who were familiar with OT interventions frequently noted improvements in mobility, pain and fatigue management, emotional stability, and social participation in daily activities. However, this positive view was not universal—some professionals questioned whether OT should be systematically included in oncology protocols, suggesting persistent inconsistency in how its relevance is perceived across institutions.

Barriers identified were multifactorial. In addition to the aforementioned lack of awareness, structural and institutional limitations—such as insufficient resources, limited funding, and underdeveloped service structures—were commonly cited. These findings are consistent with previous studies, which have reported that OT services in oncology are often constrained by systemic factors such as workforce shortages, unclear professional roles, and fragmented organizational frameworks [18,24]. Interestingly, traditional interdisciplinary barriers—such as resistance from other professionals or lack of coordination—were not dominant concerns in this study. This contrasts with earlier literature that described interprofessional tension as a common obstacle to OT integration [25,26] and may suggest that greater institutional recognition and operational support, rather than attitudinal change, could be the key to expanding OT presence in pediatric oncology settings [27].

Regional disparities were also evident, with services more concentrated mainly in urban centers such as Madrid. This reflects broader trends in the Spanish healthcare system, where access to specialized rehabilitation services often varies across autonomous communities [28]. The uneven distribution of occupational therapists across the country may be limiting access to OT in pediatric oncology, even where its need is recognized.

A consistent theme emerging from participant responses was the urgent need for specialized training in pediatric oncology for occupational therapists. This was seen as essential to ensuring effective, context-sensitive interventions and to promoting greater interdisciplinary integration. Without such training, even when therapists are available, their impact may be constrained by a lack of preparation or recognition. Previous literature has similarly highlighted that limited oncology-specific education can hinder the ability of occupational therapists to deliver targeted, evidence-based care [20]. In pediatric contexts, this challenge is amplified by the complexity of developmental needs and the emotional burden faced by both patients and families [29]. Studies have shown that therapists who receive advanced training in oncology are better prepared to address fatigue, emotional regulation, and occupational disruption in children undergoing treatment [22]. These findings suggest a notable gap in oncology-specific knowledge and training among occupational therapists in the participating centers, alongside a widely recognized need for enhanced professional development in this clinical area. This aligns with our observation that, even when OT professionals are available, their effectiveness may be limited by a lack of specialized preparation. Expanding oncology-specific education and training is, therefore, not only necessary for clinical competence but also for improving the visibility and credibility of OT within multidisciplinary teams.

### 4.1. Strengths and Limitations

This study presents several important strengths. Foremost, it addresses a highly relevant and underexplored area in the Spanish healthcare context: the integration of OT into pediatric oncology services. By applying a mixed-methods approach, the study offers both quantitative breadth and qualitative depth, capturing structural data as well as lived professional perspectives. The survey design, combining closed and open-ended items, enabled a nuanced understanding of both systemic conditions and individual experiences.

The inclusion of 42 hospital centers from 12 autonomous communities adds territorial diversity and reflects the heterogeneity of OT service provision across Spain. The qualitative component stands out as a particular strength, offering thematically grounded insights enriched with direct quotations that capture professional voices across diverse clinical settings. Furthermore, the study fills a gap in the Spanish literature and provides a foundation for future research in oncology rehabilitation.

Nonetheless, several limitations must be acknowledged. Although the sample was diverse, it was not statistically representative. Only 16 centers reported having OT services in pediatric oncology, and the qualitative findings were drawn from a relatively small number of professionals, which limits external validity. The high proportion of “Do not know” responses—exceeding 40% in key items—reflects both limited awareness of OT and restricts the robustness of the statistical analysis. Also, the high number of missing responses in some items of the survey may weaken the robustness and generalizability of the findings. This non-response bias could have affected the accuracy of the results, particularly in questions related to training, where some participants were not eligible to respond. Only 16 out of 42 centers have occupational therapy services, which reduces the volume of data available for effective impact analysis and may lead to limited or difficult-to-generalize conclusions.

No multivariate or correlation analyses were conducted in line with the study’s exploratory intent. However, this limits the ability to control for confounding variables such as hospital size, regional location, or service type. Future work could explore associations between OT integration and institutional characteristics to identify predictors of best practice and guide targeted improvements. In addition, triangulation with external institutional sources (e.g., SEHOP or RETI registries) was not performed to validate the presence of OT services reported by respondents.

Other thematic limitations include the lack of exploration into existing educational pathways or postgraduate training opportunities in pediatric oncology OT, despite training gaps being widely acknowledged by participants. The role of OT in pediatric palliative care was also underrepresented in responses and warrants further attention due to its documented relevance in this domain. Finally, the perceived impact of OT was reported subjectively without the use of validated outcome instruments such as the Canadian Occupational Performance Measure (COPM) or the Pediatric Quality of Life Inventory (PedsQL), which limits the objectivity of the findings.

Taken together, these strengths and limitations should guide the interpretation of the results and inform the design of future studies aiming to build a stronger evidence base for OT in pediatric oncology care.

### 4.2. Implications and Recommendations

The findings of this study underscore several critical implications for clinical practice, professional education, and health policy related to pediatric oncology care in Spain. Despite evidence of OT’s potential to enhance quality of life and functional outcomes in children with cancer, its integration remains inconsistent and underdeveloped. Based on the results, the following strategic actions are recommended, which include not only structural and educational reforms but also practical solutions such as specialized training programs, advocacy initiatives, and care integration protocols to strengthen OT’s institutional role (Table 5):

By pursuing these recommendations, healthcare stakeholders can strengthen the institutional role of OT and contribute to a more comprehensive, person-centered model of pediatric cancer care.

### 4.3. Future Directions

To build on these findings, future research should aim to include a broader and more representative sample of hospitals, spanning diverse regions, sizes, and service structures. Longitudinal or intervention-based designs would be particularly valuable to examine the long-term effects of OT on functional, emotional, and social outcomes in pediatric oncology patients.

There is also a pressing need to map and expand existing training opportunities in pediatric oncology OT and to assess institutional barriers to their uptake. Furthermore, advocacy efforts should prioritize increasing awareness of OT’s scope and clinical relevance among hospital administrators and multidisciplinary teams. Integrating OT more systematically into standard oncology protocols will be key to ensuring that all pediatric patients benefit from the therapeutic, rehabilitative, and psychosocial support this profession offers.

Future initiatives should also explore partnerships with professional associations, academic institutions, and regional health services to pilot implementation toolkits, training modules, and policy briefs tailored to pediatric oncology settings. Monitoring and evaluating these initiatives will be essential to inform scaling efforts and ensure sustained impact.

## 5. Conclusions

Overall, this study reveals a disconnect between the recognized benefits of OT and its limited integration in pediatric oncology care in Spain. While many professionals perceive OT as a valuable discipline, structural, educational, and institutional barriers continue to hinder its full implementation.

To improve care for pediatric cancer patients, it is essential to address these gaps through targeted interventions. This includes enhancing oncology-specific training for occupational therapists, increasing institutional recognition of the profession’s role, and ensuring equitable access to OT services across all regions. Strengthening OT’s presence within multidisciplinary teams can help optimize functional, emotional, and social outcomes for children and adolescents with cancer, thereby contributing to a more holistic and patient-centered model of care.

This disconnect underscores the importance of addressing not only systemic and institutional barriers but also the educational gaps that restrict the scope and visibility of OT. Enhanced, oncology-focused training for occupational therapists will be a critical step in promoting their effective integration and ensuring the quality of care provided to pediatric oncology patients.

In parallel, the development of national care protocols, pilot programs, and advocacy strategies can help institutionalize OT within pediatric oncology services. By advancing these complementary initiatives, the Spanish healthcare system can move toward more equitable, evidence-based, and comprehensive cancer rehabilitation for children and their families.

## Figures and Tables

**Table 1:** Summary of the key variables collected.

Variable Category	Key Aspects Assessed
Availability of OT services	Presence of OT, number of professionals, stages of treatment, settings of intervention
Training and expertise	Specific training in pediatric oncology, continuing education, knowledge of treatments
OT interventions	Areas of focus (physical, psychosocial, ADLs), personalization, interdisciplinary work
Barriers and challenges	Resource limitations, institutional support, referral issues, professional recognition
Perceived impact	Outcomes in mobility, independence, emotional well-being, patient/family satisfaction

**Table 2:** Hospital characteristics: type, location, patient volume, and patient age groups.

**Type of Hospital *n* = 42**	***F* (%)**
Public	35 (83.3)
Private	3 (7.1)
Other	4 (9.5)
**Location *n* = 42**	***F*** **(%)**
Madrid	9 (21.4)
The Basque Country	1 (2.4)
Balearic Islands	5 (11.9)
Navarra	2 (4.8)
Aragón	6 (14.3)
Canarias	2 (4.8)
Galicia	1 (2.4)
Cataluña	6 (14.3)
Andalucía	6 (14.3)
Castilla la Mancha	2 (4.8)
La Rioja	1 (2.4)
Cantabria	1 (2.4)
**Nº of Patients *n* = 42**	***F*** **(%)**
<50	11 (26.2)
50–100	15 (35.7)
101–200	6 (14.3)
>200	6 (14.3)
DK	4 (9.5)
**Age of Patients *n* = 42**	*F* (%) *
<1 year	35 (83.3) *
1–5 years	37 (88.1) *
6–12 years	38 (90.5) *
13–18 years	38 (90.5) *

Note: DK = Do not know; * These items are evaluated individually, with percentages calculated based on the total number of responses received for that specific item.

**Table 3:** Availability and features of OT services in pediatric oncology units.

**OT in the Oncology Hospital *n* = 42**	***F* (%)**
Yes	16 (38.1)
No	9 (21.4)
Dk	17 (40.5)
**Number of OT *n* = 42**	***F* ** **(%)**
1	6 (14.3)
2–5	10 (23.8)
No answer	26 (61.9)
**OT Integrated in the Multidisciplinary Team *n* = 42**	***F* ** **(%)**
Yes	11 (26.2)
No	3 (7.1)
Dk	1 (2.4)
No answer	27 (64.3)
**Years of OT in the Oncology Hospital *n* = 42**	***F*** **(%)**
<1 year	1 (2.4)
1–3 years	2 (4.8)
4–5 years	4 (9.5)
5 years	5 (11.9)
DK	4 (9.5)

Note: DK = Do not know.

**Table 4:** Training of occupational therapists in the field of pediatric oncology.

**Level of Knowledge *n* = 13**	***F* (%)**	**Sufficient Training *n* = 13**	***F* (%)**
High	3 (7.1)	Yes	4 (9.5)
Low	3 (7.1)	No	2 (4.8)
No have	3 (7.1)	DK	7 (16.7)
DK	4 (9.5)	No answer	29 (69)
No answer	29 (69)		
**Training of OT *n* = 13**	***F* (%)**	**Participating in Training *n* = 12**	***F* ** **(%)**
Yes	5 (11.9)	Yes	4 (9.5)
No	6 (14.3)	No	3 (7.1)
DK	2 (4.8)	DK	5 (11.9)
No answer	29 (69)	No answer	30 (71.4)
**They Should Receive More Training *n* = 32**	***F*** **(%)**		
Yes	25 (59.5)		
No	0 (0)		
DK	7 (16.7)		
No answer	10 (23.8)		

Note: DK = Do not know.

**Table 5:** Strategic recommendations for integrating OT in pediatric oncology care in Spain.

**1. Develop Specialized Training Programs**
Create postgraduate certification programs focused on pediatric oncology rehabilitation, in collaboration with universities, professional colleges, and oncology units. Integrate oncology modules into existing occupational therapy curricula to ensure early exposure and competency in cancer care. Offer continuing education workshops and interdisciplinary training seminars for in-service professionals, including OT-specific sessions at national oncology or rehabilitation conferences.
**2. Implement Institutional Integration Protocols**
Establish standardized care pathways that formally incorporate OT into multidisciplinary pediatric oncology teams, from diagnosis through survivorship and palliative care. Define clear referral criteria and workflows to ensure timely access to OT services for eligible patients. Develop internal hospital guidelines for interdisciplinary collaboration, including regular case reviews and care planning meetings involving OT professionals.
**3. Strengthen Interprofessional Collaboration and Recognition**
Promote awareness among hospital administrators, medical professionals, and policymakers about the role and benefits of OT in pediatric oncology. Include OT representation in oncology committees, pediatric tumor boards, and protocol development teams to increase visibility and influence in institutional decision-making. Support interdisciplinary research initiatives that include OT as a central focus or partner, fostering collaborative evidence generation.
**4. Advance Policy and Advocacy Efforts**
Collaborate with national and regional health authorities to include OT services in public pediatric oncology funding models and workforce planning. Advocate for policy reforms that establish minimum standards for OT provision in cancer care settings, particularly for pediatric populations. Support efforts by professional associations to raise awareness about OT’s role in oncology rehabilitation through media campaigns, policy briefs, and stakeholder engagement.
**5. Promote Equitable Service Distribution**
Design and implement regional pilot programs in underserved areas to demonstrate the impact of OT on pediatric cancer outcomes. Create mobile or telehealth-based OT services to support rural hospitals and increase accessibility for families unable to travel to larger centers. Encourage inter-hospital collaboration and knowledge sharing to standardize care across autonomous communities.
**6. Standardize Outcomes and Data Collection**
Integrate validated outcome measures—such as the Canadian Occupational Performance Measure (COPM) and the Pediatric Quality of Life Inventory (PedsQL)—into routine clinical practice and research. Encourage centers to report and share implementation data to build a national evidence base for OT in pediatric oncology.

## Data Availability

The data supporting the findings of this study are available from the corresponding author upon reasonable request. Due to privacy and ethical restrictions, the datasets are not publicly accessible.

## Supplementary Materials

The following supporting information can be downloaded at: https://www.mdpi.com/article/10.3390/healthcare13141737/s1, Supplementary Materials S1: Survey.

## Abbreviations

The following abbreviations are used in this manuscript:

OT	Occupational Therapy
CEICA	Clinical Research Ethics Committee of Aragón
DK	Don’t know
COPM	Canadian Occupational Performance Measure
PedsQL	Pediatric Quality of Life Inventory
