# Supplementary material for: Exploring the Integration of Occupational Therapy in Pediatric Oncology Care in Spain: A Descriptive Study
Source: Healthcare (Basel). 2025 Jul 18;13(14):1737. doi: 10.3390/healthcare13141737 (PMC12295375; doi:10.3390/healthcare13141737)
Supplement: Supplementary file 1 [file healthcare-13-01737-s001.zip › healthcare-3733240-supplementary.pdf]

## Supplementary file S1: Survey

### Section 1: General information about the center/unit

1. What type of institution do you work in?
   • Public hospital
   • Private hospital
   • Public-private partnership hospital
   • Other (please specify): ___________
2. Where is your center/unit located?
   • Mainland Spain
   • Balearic Islands
   • Canary Islands
   • Other (please specify): ___________
3. In which city is your health center or hospital located?
   (Open-ended question)
   Answer: ___________
4. In which province is your health center or hospital located?
   (Open-ended question)
   Answer: ___________
5. How many pediatric cancer patients does your pediatric oncology center/unit treat annually?
   • Fewer than 50
   • 50–100
   • 101–200
   • More than 200
   • I don't know / I don't have this information
6. What is the age range of the patients treated in your pediatric oncology center/unit? (You may select more than one option)
   • Under 1 year old
   • 1–5 years old
   • 6–12 years old
   • 13–18 years old

### Section 2: Availability of Occupational Therapy in pediatric oncology

1. Does your center/unit offer Occupational Therapy services for pediatric cancer patients?
   • Yes
   • No
   • I'm not sure
2. Since when has Occupational Therapy been offered in your pediatric oncology center/unit?
   • Less than 1 year ago
   • 1–3 years ago
   • 4–5 years ago
   • More than 5 years ago
   • This service is not offered
   • I'm not sure
   • Not applicable (Occupational Therapy services are not offered)
3. How many Occupational Therapy professionals work in your pediatric oncology center/unit?
   • 1

• 2–5
• More than 5
• This service is not provided

4. Is Occupational Therapy integrated into the multidisciplinary team for pediatric oncology patient care?
   • Yes
   • No
   • I don't know
   • Not applicable (Occupational Therapy services are not offered)
5. At what stage of cancer treatment is Occupational Therapy provided to patients? (You may select more than one option)
   • During diagnosis and initial treatment
   • During active treatment (chemotherapy, radiotherapy, etc.)
   • Post-treatment rehabilitation phase
   • During remission or follow-up phase
   • Palliative phase
   • Not applicable (Occupational Therapy services are not offered)
   • Other (please specify): ___________
6. In what settings is Occupational Therapy provided to pediatric cancer patients? (You may select more than one option)
   • In the hospital (inpatients)
   • Outpatient clinics
   • Palliative care unit
   • Ambulatory follow-up
   • Post-surgical rehabilitation
   • Day hospital
   • Specialized treatment units
   • Other (please specify): ___________
   • Not applicable (Occupational Therapy services are not offered)
7. What factors determine whether a patient receives Occupational Therapy? (You may select more than one option)
   • Patients with rare or complex oncological diseases
   • Patients with significant physical limitations (e.g., reduced mobility, disability)
   • Patients with psychological or emotional issues
   • All patients
   • I don't know / I'm not sure
   • Other (please specify): ___________
   • Not applicable (Occupational Therapy services are not offered)
8. Is Occupational Therapy available to all pediatric cancer patients in your center/unit or only to certain groups?
   • Available to all patients
   • Only to patients with certain conditions (please specify): ___________
   • Not available (Occupational Therapy services are not offered)
9. In your center/unit, is access to Occupational Therapy services influenced by gender factors?
   • Yes, girls have more access
   • Yes, boys have more access
   • No gender differences
   • Other factor (please specify): ________
   • I don't know
   • Not applicable (Occupational Therapy services are not offered)
10. Who coordinates Occupational Therapy care in your pediatric oncology center/unit?
    • A specific Occupational Therapy coordinator
    • The head of the pediatric oncology unit
    • Another professional (please specify): ___________

• There is no specific coordination
• Not applicable (Occupational Therapy services are not offered)

11. Is there any kind of coordination in your center/unit between Occupational Therapy and other specialties or services (psychology, physiotherapy, social work, etc.)?
    • Yes, there is active and frequent coordination
    • Yes, there is coordination, but sporadically
    • No, there is no coordination
    • I don't know
    • Not applicable (Occupational Therapy services are not offered)

**Section 3: Training and education of Occupational Therapists**

1. Do the occupational therapists in your center/unit have specific training in pediatric oncology, including aspects related to treatment, side effects, and the specific needs of pediatric cancer patients?
   • Yes
   • No
   • Partially (please specify): ___________
   • Not applicable (Occupational Therapy services are not offered)
2. Do you consider the current training of occupational therapists in your center/unit sufficient to address the needs of pediatric cancer patients?
   • Yes, it is sufficient
   • No, it is insufficient
   • I don't know
   • Not applicable (Occupational Therapy services are not offered)
3. Do occupational therapists in your center/unit participate in ongoing professional development programs in the field of pediatric oncology?
   • Yes, regularly (seminars, conferences, etc.)
   • Yes, occasionally
   • No
   • I don't know
   • Not applicable (Occupational Therapy services are not offered)
4. What is the level of knowledge that occupational therapists in your center/unit have about pediatric oncology treatments (chemotherapy, radiotherapy, transplants, etc.)?
   • Very high knowledge
   • Moderate knowledge
   • Low knowledge
   • They have no specific knowledge of these treatments
   • I'm not sure
   • Not applicable (Occupational Therapy services are not offered)
5. Do you think occupational therapists should receive more training in pediatric oncology?
   • Yes
   • No
   • I have no opinion
6. What type of training do you consider most relevant for occupational therapists working with pediatric oncology patients?
   (Open-ended question)
   Answer: ___________

7. What do you think are the main areas for improvement in the training of occupational therapists in the field of pediatric oncology?
   (Open-ended question)
   Answer: ___________

**Section 4: Occupational Therapy interventions in pediatric oncology**

1. What is the main goal of Occupational Therapy for pediatric oncology patients in your center/unit? (You may select multiple options)
   • Improve mobility and physical function
   • Improve activities of daily living (ADLs)
   • Support psychosocial and emotional adaptation
   • Support adaptation to the environment and use of assistive devices (wheelchairs, prosthetics, etc.)
   • Increase participation in social and recreational activities
   • Other (please specify): ___________
   • Not applicable (Occupational Therapy services are not offered)
2. What types of Occupational Therapy interventions are offered to pediatric cancer patients in your center/unit? (You may select multiple options)
   • Physical rehabilitation (improving strength, mobility, motor skills)
   • Psychosocial support (coping strategies, emotional regulation)
   • Training and adaptation of daily living activities (use of devices, environmental modification)
   • Environmental adaptation
   • Training in social and communication skills
   • Prevention of long-term complications (chronic fatigue, physical disabilities)
   • Recreational and leisure activities
   • Pain management
   • Cognitive rehabilitation
   • Other (please specify): ___________
   • Not applicable (Occupational Therapy services are not offered)
3. How are Occupational Therapy interventions prioritized in your pediatric oncology center/unit? (You may select multiple options)
   • Based on the severity of the patient's medical condition
   • Based on the patient's functional needs (e.g., mobility, daily living skills)
   • Based on the stage of cancer treatment (chemotherapy, recovery, palliative care)
   • There is no specific prioritization criteria
   • Other (please specify): ___________
   • Not applicable (Occupational Therapy services are not offered)
4. Have you noticed any differences in the type of Occupational Therapy interventions provided based on the gender of the patients?
   • Yes, girls receive more psychosocial and emotional support
   • Yes, boys receive more physical rehabilitation
   • No gender differences in interventions
   • I don't know
   • Not applicable (Occupational Therapy services are not offered)
5. What types of assessment tools does your Occupational Therapy team use to evaluate the needs and progress of pediatric oncology patients? (You may select multiple options)
   • Standardized questionnaires (e.g., COPM, PEDI, etc.)

- Functional assessments (such as mobility or ADL tests)
- Quality of life assessments (physical, emotional, social)
- Fatigue and/or pain assessments
- Interviews and subjective assessments with patients and families
- Direct observation of the patient
- No standardized assessments are conducted
- Other (please specify): ___________
- Not applicable (Occupational Therapy services are not offered)

6. To what extent are Occupational Therapy interventions in your center/unit personalized according to the individual characteristics of each patient (age, type of cancer, treatments received, etc.)?
   - Fully personalized
   - Largely personalized
   - Minimally personalized
   - Interventions are not personalized
   - I'm not sure
   - Not applicable (Occupational Therapy services are not offered)
7. Are Occupational Therapy interventions conducted in collaboration with other disciplines (psychology, physical therapy, social work, etc.) in your center/unit?
   - Yes, always as part of a multidisciplinary team
   - Yes, but not always
   - No, there is no multidisciplinary collaboration
   - I don’t know
   - Not applicable (Occupational Therapy services are not offered)
8. In your center/unit, is there any type of follow-up or continuity of Occupational Therapy interventions after the patient completes cancer treatment?
   - Yes, regular follow-up during remission
   - Yes, occasional follow-up as needed
   - No, no post-treatment follow-up
   - I don’t know
   - Not applicable (Occupational Therapy services are not offered)
9. Do you consider Occupational Therapy interventions in your unit effective in improving the quality of life of pediatric cancer patients?
   - Yes, to a great extent
   - Yes, to a moderate extent
   - No, the interventions are not effective
   - I don’t know
   - Not applicable (Occupational Therapy services are not offered)
10. In your opinion, what is the most beneficial Occupational Therapy intervention for pediatric cancer patients?
    (Open-ended question)
    Answer: ___________

**Section 5: Barriers and challenges in the integration of Occupational Therapy**

1. What are the main obstacles to integrating Occupational Therapy into pediatric oncology care in your center/unit? (You may select multiple options)
   - Lack of resources (personnel, space, materials)
   - Lack of financial resources (insufficient budget)
   - Lack of time due to workload

• Lack of awareness of the importance of Occupational Therapy
• Lack of recognition of the value of Occupational Therapy by the multidisciplinary team
• Lack of coordination between different healthcare services
• Lack of evidence on the effectiveness of Occupational Therapy in this context
• Emotional or psychological barriers in patients (fear, treatment refusal)
• Resistance or lack of support from other professionals on the multidisciplinary team
• Lack of training among healthcare staff
• Organizational or administrative barriers
• Difficulty involving families in treatment
• There are no obstacles
• Other (please specify): ___________

2. Do you think Occupational Therapy in pediatric oncology receives the necessary support from hospital administration or health managers?
   • Yes, there is always institutional support
   • Yes, but the support is limited
   • No, there is not enough support
   • I don't know
   • Not applicable (Occupational Therapy services are not offered)
3. Are there delays or difficulties in referring patients to Occupational Therapy?
   • Yes, delays are frequent
   • Yes, there are occasional delays
   • No, there are no delays
   • I don't know
   • Patients are not referred
4. Do you consider that the integration of Occupational Therapy into the treatment of pediatric cancer patients is sufficiently recognized within the multidisciplinary team in your center/unit?
   • Yes, it is fully recognized
   • Yes, but it could be more recognized
   • No, it is not sufficiently recognized
   • I don't know
   • Not applicable (Occupational Therapy services are not offered)
5. In your center/unit, does the Occupational Therapy team have enough time to provide quality interventions for all pediatric cancer patients?
   • Yes, the time is adequate
   • No, the time is insufficient to attend to all patients
   • No, the time is insufficient to offer quality interventions
   • I don't know
   • Not applicable (Occupational Therapy services are not offered)
6. Do you believe pediatric cancer patients and their families in your center/unit have sufficient knowledge about the benefits of Occupational Therapy in cancer treatment?
   • Yes, they are well informed
   • No, they are not well informed
   • I don't know
   • Not applicable (Occupational Therapy services are not offered)
7. What measures do you think could be taken to improve the integration of Occupational Therapy in pediatric oncology care?
   (Open-ended question)
   Answer: ___________

**Section 6: Impact and evaluation of Occupational Therapy**

1. Do you consider Occupational Therapy to be an essential service for pediatric cancer patients in your center/unit (essential in the sense that it significantly contributes to improving patients' quality of life or recovery)?
   • Yes
   • No
   • I don't know
   • Not applicable (Occupational Therapy services are not offered)
2. How is the impact of Occupational Therapy on the quality of life of pediatric cancer patients evaluated in your center/unit?
   • Very positive
   • Positive
   • Neutral
   • Negative
   • Very negative
   • No evaluation is conducted
   • Not applicable (Occupational Therapy services are not offered)
3. In which areas do you think Occupational Therapy has the greatest impact on pediatric oncology patients in your center/unit?
   (You may select multiple options)
   • Improvement in mobility and physical strength
   • Recovery of daily living skills (e.g., dressing, eating, personal hygiene)
   • Improvement in emotional and psychosocial wellbeing (coping, emotional well-being)
   • Better social integration (interaction with other children, participation in recreational activities)
   • Prevention of long-term complications (fatigue, chronic pain, disability)
   • Improvement in communication and social skills
   • Improvement in cognition and academic skills
   • Other (please specify): ___________
   • Not applicable (Occupational Therapy services are not offered)
4. Do you think Occupational Therapy interventions in your center/unit have a different impact based on the gender of the patients?
   • Yes, interventions have a greater impact on boys
   • Yes, interventions have a greater impact on girls
   • No, the impact is the same regardless of gender
   • I don't know
   • Not applicable (Occupational Therapy services are not offered)
5. Do you consider that Occupational Therapy helps patients in your center/unit to maintain or improve their level of independence during cancer treatment?
   • Yes, significantly
   • Yes, to some extent
   • No, it does not improve independence
   • I don't know
   • Not applicable (Occupational Therapy services are not offered)
6. Do you believe early Occupational Therapy intervention improves long-term outcomes for pediatric cancer patients in your center/unit?
   • Yes, it significantly improves long-term quality of life and functioning
   • Yes, it moderately improves long-term quality of life and functioning
   • No, it has no long-term impact

• I don't know
• Not applicable (Occupational Therapy services are not offered)

7. How do you perceive the impact of Occupational Therapy in reducing the side effects of cancer treatment (e.g., fatigue, pain, loss of motor skills) in the patients in your center/unit?
   • Very positive – it considerably reduces side effects
   • Positive – it reduces some side effects
   • Neutral – it does not have a major impact
   • Negative – it seems to worsen side effects
   • I don't know
   • Not applicable (Occupational Therapy services are not offered)
8. What changes or improvements have you observed in the patients in your center/unit following Occupational Therapy interventions during cancer treatment? For example, improvements in mobility, quality of life, daily living activities, emotional well-being, etc.
   (Open-ended question)
   Answer: ___________
9. How do you assess patient and family satisfaction with Occupational Therapy interventions in your center/unit?
   • Very high satisfaction
   • High satisfaction
   • Moderate satisfaction
   • Low satisfaction
   • I don't know
   • Not applicable (Occupational Therapy services are not offered)
10. Do you think Occupational Therapy should be part of the standard treatment protocols in pediatric oncology in your center/unit?
   • Yes
   • No
   • I'm not sure

**Section 7: Opinions and suggestions**

1. In your experience, what aspects of Occupational Therapy could be improved to increase its effectiveness in pediatric oncology treatment? For example: therapist training, integration into the multidisciplinary team, availability of resources, frequency of interventions, etc.
   (Open-ended question)
   Answer: ___________
2. Would you like to add any additional comments regarding the role of Occupational Therapy in pediatric oncology?
   (Open-ended question)
   Answer: ___________
